# Case report of anti-N-methyl-D-aspartate receptor encephalitis in a middle-aged woman with a long history of major depressive disorder

**DOI:** 10.1186/s12888-017-1477-x

**Published:** 2017-08-31

**Authors:** Xia Rong, Zhenzhen Xiong, Bingrong Cao, Juan Chen, Mingli Li, Zhe Li

**Affiliations:** 1 Department of Rheumatology, West China Hospital, Sichuan University, Chengdu, Sichuan 610041 China; 2 School of Nursing, Chengdu Medical College, Chengdu, Sichuan 610083 China; 3 Mental Health Center and Psychiatric Laboratory, West China Hospital, Sichuan University, Chengdu, Sichuan 610041 China; 4 The Mental Rehabilitation Center, Karamay Municipal People’s Hospital, Karamay, Xinjiang 830054 China

**Keywords:** Anti-N-methyl-D-aspartate receptor encephalitis, Depressive disorder, Differential diagnosis, Fluctuating course

## Abstract

**Background:**

Anti-N-methyl-D-aspartate receptor (NMDAR) encephalitis is an autoimmune disease involving antibodies against the NR1 subunits of NMDARs. The disease shows variable clinical presentation, and involves new-onset acute psychotic symptoms, making it difficult to differentiate from major depressive disorder with psychotic symptoms. Potential associations between this autoimmune disorder and onset or progression of major depressive disorder remains unclear.

**Case presentation:**

We present a rare case of a patient who had both major depressive disorder and anti-NMDAR encephalitis and in whom the encephalitis initially went undetected. The patient had been suffering from depressive disorder for more than 6 years without any treatment, when she was hospitalized for new-onset psychotic symptoms. She was initially diagnosed only with major depressive disorder with psychotic symptoms, but antipsychotics did not alleviate symptoms and the patient’s psychiatric course began to fluctuate rapidly. Anti-NR1 IgG autoantibodies were detected in cerebrospinal fluid, and the combination of immunotherapy and antipsychotics proved more effective than antipsychotics alone. The patient was then also diagnosed with anti-NMDAR encephalitis.

**Conclusions:**

Our case suggests that clinicians should consider anti-NMDAR encephalitis when a patient with depressive disorder shows sudden fluctuations in psychiatric symptoms. It also highlights the need for research into possible relationships between anti-NMDAR encephalitis and major depressive disorder.

## Background

First described in 2007 by Dalmau and colleagues, anti-N-methyl-D-aspartate receptor (NMDAR) encephalitis is a synaptic autoimmune condition in which anti-NMDAR antibodies are detectable in serum or cerebrospinal fluid (CSF) [1]. Prevalence of this disease is unclear, but mortality is known to be as high as 25% [2]. While analysis of more than 500 cases indicates that the disease shows variable clinical presentation, many patients show acute-onset psychiatric symptoms [3–5]. The major symptoms may include delusions, hallucinations, mania, agitation, abnormal behaviour or cognitive dysfunction, speech dysfunction, seizures, movement disorder, decreased level of consciousness, autonomic and breathing instability [6].

Dalmau et al. [4] and Maneta et al. [5] have described a staged presentation comprising an early (prodrome) phase, a middle phase involving psychiatric manifestations and a late phase involving physical symptoms. Analysis of a case series of 100 individuals showed that in the weeks leading up to acute presentation, 86 experienced a non-specific prodrome phase involving low-grade fever, headache, and respiratory or gastrointestinal symptoms [2]. At 5 days to 2 weeks after prodrome onset, one or more of the following psychiatric symptoms developed: agitation, delusions, hallucinations, mania, and cognitive dysfunction. Most patients ultimately entered an unresponsive phase involving seizure, motor dysfunction (especially orofacial dyskinesia), autonomic instability or hypoventilation.

Anti-NMDAR encephalitis has been observed in patients of all ages, though young women appear to be disproportionately affected [3, 7, 8]. Two case series suggested co-morbidity with ovarian pathology in 59 of 100 patients (59%) [2] and in 9 of 34 patients (26%) [9]. These case series did not include patients younger than 18, so the actual rates of co-morbidity may be different.

Most patients initially seek help from a psychiatrist, but the disorder is difficult to recognize because psychosis is evident but acute-onset psychiatric episodes are typically absent. Schizophrenia has been reported in several patients who later turned out to have encephalitis [10]. To our knowledge, only one case has been reported in which the disorder was detected in a patient (16-year-old girl) previously diagnosed with severe depression [11].

## Case presentation

Here, we present a 52-year-old Chinese woman. 6 years before her admission, she began to feel a depressive mood, inferiority and desperation, as well as insomnia, retardation, and social withdrawal. She lacked interest in doing anything and rarely felt happy. She lost any desire to prepare meals for her family and complained constantly. She reported having thoughts about suicide but never attempted it. She and her family did not act on these symptoms, so the patient never sought or received medical treatment. One month before her admission, the depressed mood exacerbated. She felt hopeless, worthlessness, guilt, and had recurrent suicidal ideation. At the same time, she developed into delusions, feelings of reference and persecution, and auditory hallucinations in the form of commentary, repetitive speaking about inappropriate sexual relations, death and the dead body. She also exhibited abnormal behaviors including irrational laughter, yelling, cursing and praising God. Although her consciousness was not disturbed, she occasionally failed to recognize her husband or daughters. Her appetite and sleep were poor.

She underwent hysterectomy, bilateral salpingo-oophorectomy and postoperative radio-chemotherapy to treat cervical carcinoma 6 years ago. The patient reported never drinking or smoking, and she was allergic to pollen. She had worked as a kindergarten teacher, but she stopped working after giving birth. Her husband did not live with the patient and daughters. She had no family history of mental disorders.

Her vital signs were stable, and no abnormal physical or neurological signs were detected at admission. Blood and urine tests were routine, blood glucose and liver and renal functions were normal, and no evidence of infection was found. Antithyroglobulin antibody was 273.80 IU/ml (reference value<115), antithyroperoxidase antibody was 596.10 IU/ml (reference value<34), free triiodothyronine was 3.35 pmol/L (reference value 3.60–7.50). Electroencephalography (EEG), electrocardiogram, and transcranial Doppler ultrasound results were normal. Antinuclear antibody (ANA) was detected in serum at a titer of 1:1000, while antibodies against Extractable nuclear antigen (ENA) or cardiolipin were not detected in serum. Brain MRI revealed several small ischemic foci in bilateral frontal lobes. The patient can’t finish the cognitive testing due to non-cooperation when admission. She and her family members denied any abuse of alcohol or illicit substances. Her family history was negative for mental illness.

The patient was diagnosed with severe major depressive disorder with psychotic features, based on the Structured Clinical Interview in the 4th edition of the Diagnostic and Statistical Manual of Mental Disorders (DSM-IV). She was treated with venlafaxine (75 mg every morning, increased 5 days later to 150 mg), lorazepam (0.5 mg every night), olanzapine (5 mg every morning, 15 mg every night), and electroconvulsive therapy (on two occasions). 5 days later, she slept well, but her mood still depressed and presented delusions of persecution, auditory hallucinations, abnormal behaviors continued. After she had spent one week in the hospital, her condition suddenly worsened: she began to talk nonsense loudly, she felt disoriented, and she did not recognize family members or her environment. She became restless and drank water repeatedly throughout the night. The next morning she suddenly stopped speaking. These symptoms continued to alternate over the course of each day.

This rapidly and dramatically changing disease course led us to organize a consultation with the neurology department. Neurological examination gave no remarkable findings, and lumber puncture was advised. CSF analysis showed no remarkable abnormalities in pressure, appearance, cells, proteins, glucose, chloride, IgG synthesis rate, or results of smear culturing for bacteria, tubercle bacilli, and fungi. The CSF was assayed for autoimmune encephalitis antibodies at Peking Union Medical College Hospital. Cell based assay was used to detect the antibodies of NMDAR, CASPR2, AMPA1-R, AMPA2-R, LGI1 and GABA2-R, and immunoblotting was used to detect the antibodies of Hu, Ri, Yo, PNMA2 (Ma2/Ta), and CV2/CRMP2 [6, 12]. The results indicated the presence of specific IgG antibodies against NR1 in NMDAR at a titer of 1:100 (Cut-off value 1:10). The other antibodies including CASPR2, AMPA1-R, AMPA2-R, LGI1, GABA2-R, Hu, Ri, Yo, PNMA2 (Ma2/Ta), and CV2/CRMP2 were negative. The patient was diagnosed with concurrent anti-NMDAR encephalitis and severe major depressive disorder with psychotic features.

At 15 days after hospitalization, the patient was transferred to the neurology department for further treatment. She was given intravenous methylprednisolone (1000 mg daily for 5 days) and immunoglobulin (20 g daily for 5 days), as well as venlafaxine (75 mg every morning), lorazepam (0.5 mg every night) and olanzapine (5 mg every night). During 2 weeks of treatment, her disorientation, behavioral disturbances, hallucinations, and delusions gradually disappeared. She was discharged still in a depressive mood; she was not talkative and showed obvious social withdrawal behavior.

2 months later, she was re-admitted to the neurology department as normal follow-up. Her emotional state, appetite and sleep were good. No cognitive or behavioral abnormalities were found. Lumber puncture was conducted a second time, and CSF analysis revealed the presence of specific IgG antibodies against NR1 in NMDAR at a reduced titer of 1:10. She was prescribed oral medications (venlafaxine 75 mg every morning, lorazepam 0.5 mg every night, olanzapine 5 mg every night) and followed up on an outpatient basis.

## Discussion

To our knowledge, this is the second case report of anti-NMDAR encephalitis in a patient with a clear diagnosis of major depressive disorder [11]. The diagnosis of anti-NMDAR encephalitis is supported by the high titer of specific IgG anti-NR1 antibodies in CSF, and the observation that the patient’s severe psychiatric symptoms markedly improved after the first treatment course of high-dose intravenous methylprednisolone and immunoglobulin.

The occurrence of both anti-NMDAR encephalitis and tumor which overwhelmingly found to be ovarian teratoma has been observed before [2, 9, 13, 14], but whether the two conditions are linked is unknown. The treatment options for anti-NMDAR encephalitis include tumor removal [3]. As for our patient, after removing the tumor, she became presenting depressed mood for 6 years, and the psychotic symptoms developed one month before her admission. The patient never sought or received medical treatment before, so antibodies in CSF can’t be detected 6 years ago. The relationship between cervical carcinoma and the onset of anti-NMDAR encephalitis in our patient remained unclear. In addition, after immunotherapy, her psychotic symptom remitted, but depressive mood still existed. Anti-NMDAR encephalitis has also been reported to present and evolved with isolated psychiatric disturbance [15], and its course can fluctuate suddenly [11]. So, the dual diagnosis of severe major depressive disorder with psychotic features and anti-NMDAR encephalitis can explain our patient’s symptoms better than either diagnosis on its own.

Thyroid hormone and thyroid autoantibodies existed in our patient. The possibility of Hashimoto encephalitis should be considered [6]. But, absence of neurological signs and seizure and a normal EEG are not features of Hashimoto encephalitis. Patients with anti-NMDAR encephalitis had abnormal EEG, such as focal or diffuse slow or disorganised activity, epileptic activity, or extreme delta brush [6], but our patient showed a normal EEG. A 33-year-old woman with anti-NMDAR encephalitis also demonstrated normal EEG [8]. The previous studies indicated that non-specific findings have been described in EEG [10, 16]. So, diagnosis of anti-NMDAR encephalitis remains a challenge according to single laboratory study.

The acute psychosis in anti-NMDAR encephalitis has more recently been associated with IgG antibodies against the NR1a, NR2a and NR2b subunits of NMDAR [17, 18]. Anti-NMDAR antibodies are believed to inhibit receptor activity in presynaptic gamma-aminobutyric acid-ergic neurons of the thalamus and frontal cortex, de-inhibiting postsynaptic glutamatergic neurons and thereby dysregulating glutamatergic and dopaminergic signaling in these brain regions [19]. NMDAR hypofunction may complement the dopaminergic model for explaining the pathogenesis of schizophrenia.

Analysis of serum samples from 70 patients with major depressive disorder showed that 2 were positive for anti-NMDAR encephalitis antibodies [17]. That study, together with the present case report, suggests that anti-NMDAR autoimmunity may be relevant to major depressive disorder, but the details of this association remain unclear. Interestingly, the repertoire of anti-NMDAR antibody subtypes in the study of 70 patients differed from the specific anti-NR1a IgG autoantibodies detected in our patient. This suggests a potentially more complex relationship between anti-NMDAR encephalitis and major depressive disorder.

When treating patients with anti-NMDAR encephalitis, it may be useful to monitor titers of specific anti-NMDAR antibodies in order to assess therapeutic efficacy [20]. We could not do this during treatment or follow-up because our hospital was not equipped for such tests, and the tests that we requested at Peking Union Medical College Hospital provide only qualitative results. At the last follow-up with our patient at 8 weeks after discharge, she reported being in a reasonably good mood with no signs of depression or hallucinations, and her behavior was stable and normal. It would be interesting to follow-up over a longer period in order to examine long-term outcomes and confirm the success of diagnosis and treatment.

## Conclusions

Our case report highlights the need for clinicians to consider the possibility of anti-NMDAR encephalitis after a long-term diagnostic history of depressive disorder, especially in patients presenting with new-onset acute psychotic symptoms and alternated so abruptly, and presenting with disorientation, even no specific findings on routine neuroimaging, EEG, or CSF analysis. It also highlights the need for further research into potential associations between this autoimmune disorder and onset or progression of major depressive disorder and schizophrenia.

## Abbreviations

ANA: Antinuclear Antibody
CSF: Cerebrospinal Fluid
DSM: Diagnostic and Statistical Manual of Mental Disorders
EEG: Electroencephalography
ENA: Extractable Nuclear Antigen
MRI: Magnetic Resonance Imaging
NMDAR: N-methyl-D-aspartate receptor
